# A Non-linear Model Predictive Control Based on Grey-Wolf Optimization Using Least-Square Support Vector Machine for Product Concentration Control in l-Lysine Fermentation

**DOI:** 10.3390/s20113335

**Published:** 2020-06-11

**Authors:** Bo Wang, Muhammad Shahzad, Xianglin Zhu, Khalil Ur Rehman, Saad Uddin

**Affiliations:** 1 School of Electrical and Information Engineering, Jiangsu University, Zhenjiang 212013, China; wangbo@ujs.edu.cn (B.W.); zxl4390@126.com (X.Z.); 5102180315@stmail.ujs.edu.cn (K.U.R.); 2 School of Mechanical Engineering, Jiangsu University, Zhenjiang 212013, China; 5102180314@stmail.ujs.edu.cn

**Keywords:** model predictive control, machine learning, grey-wolf optimization, least-square support vector machine, l-Lysine fermentation

## Abstract

l-Lysine is produced by a complex non-linear fermentation process. A non-linear model predictive control (NMPC) scheme is proposed to control product concentration in real time for enhancing production. However, product concentration cannot be directly measured in real time. Least-square support vector machine (LSSVM) is used to predict product concentration in real time. Grey-Wolf Optimization (GWO) algorithm is used to optimize the key model parameters (penalty factor and kernel width) of LSSVM for increasing its prediction accuracy (GWO-LSSVM). The proposed optimal prediction model is used as a process model in the non-linear model predictive control to predict product concentration. GWO is also used to solve the non-convex optimization problem in non-linear model predictive control (GWO-NMPC) for calculating optimal future inputs. The proposed GWO-based prediction model (GWO-LSSVM) and non-linear model predictive control (GWO-NMPC) are compared with the Particle Swarm Optimization (PSO)-based prediction model (PSO-LSSVM) and non-linear model predictive control (PSO-NMPC) to validate their effectiveness. The comparative results show that the prediction accuracy, adaptability, real-time tracking ability, overall error and control precision of GWO-based predictive control is better compared to PSO-based predictive control.

## 1. Introduction

The invention and advancement of modern computationally fast microprocessors have paved the path for Model Predictive Control (MPC). Recently, MPC has become one of the efficient predictive control algorithms in large scale applications such as aerospace systems [1], plastic industry [2], wastewater treatment plant [3], power electronics industry [4] and many others. It can handle multi-process variables and incorporate practical constraints on these variables. The basic elements of MPC are: process model, cost function and optimization algorithm. The process model plays a key role in the performance of MPC. It should encompass the precise dynamics of the process. In most applications, linearized models are used in MPC. However, practical processes exhibit severe non-linearity that cannot be captured by linear models. In addition, these linear models cannot cover a wide range of operating conditions. Artificial intelligence-based models are famous for their self-learning and non-linear modeling ability, and have attracted many researchers to model these non-linear behaviors such as artificial neural network (ANN) and support vector machine (SVM) [5,6].

l-Lysine is the second most produced amino acid in the world. An estimated global market of l-Lysine is 2.2 million tons per year, which is growing at the rate of 10% per year [7]. It is mainly used in food, animal feed, pharmaceuticals and cosmetics industries. This increasing demand in global market compels industries to look for alternatives to enhance the productivity instead of expanding the physical capacity of plants, which is expensive and time consuming. One of the best ways to boost the productivity is to monitor and control product concentration. An excessive increase of product in reactor causes osmotic stress or catabolic repression for bacteria during cultivation [8]. Temperature, pH, initial substrate concentration, air flow rate and agitation rate are five paramount factors to enhance and control product concentration [9]. However, l-Lysine fermentation is a highly non-linear process and product concentration cannot be directly measured in real time using physical sensors in the fermentation process. Some costly off-line analysis methods such as dry weight method, ninhydrin colorimetric method and optical density method are used to measure product concentration, but these methods have limitations, such as large time delay and high infection rate. Therefore, off-line lab analyzers cannot meet the requirements of real-time control of the fermentation process with these limitations. To solve the above mentioned problems, machine-learning-based prediction models [10] have been effectively used by the researchers [11].

Machine-learning-based prediction models construct inferential mathematical models by making use of easily measurable variables (for example, pH, temperature and dissolved oxygen) obtained from physical sensors and predict the unmeasurable key variables (product concentration) [12]. The successful implementation of these prediction models has revolutionized the fermentation industry. Researchers have introduced many data-driven prediction models to model fermentation process for different objectives. ANN is exploited to design a prediction model for bioethanol production by defining optimal number of hidden layers and hidden units [13]. However, if the specific structure of network is not known, ANN loses its generalization ability to model non-linear regression problems and suffers from overfitting problem [14]. SVM, which is based on structural risk minimization problem, has successfully resolved the aforementioned problem using simple statistical learning theory. It is used to solve many industrial applications, such as a modelling method based on SVM is proposed for Glutamic acid fermentation to predict product concentration [15]. Unfortunately, huge time cost for training and curse of dimensionality limits usefulness of SVM in many applications [16]. Least-square support vector machine (LSSVM) alleviates this problem by converting convex Quadratic Programming (QP) problem in SVM to a system of linear equations. In this way, LSSVM provides fast training speed and efficiently finds global optimum solution if the parameters are selected carefully [17].

In this study, LSSVM is selected as a prediction model to measure product concentration of l-Lysine fermentation process. To improve the prediction accuracy and robustness of LSSVM, two model parameters, namely kernel width ‘σ’ and regularization factor ‘*g*’ must be optimized. A very large value of penalty parameter ‘*g*’ would lead to remarkably high accuracy on training data but less accuracy on test data, while less value makes the model less functional resulting in poor performance. In addition, an excessively large value of kernel width control factor ‘σ’ inflicts overfitting problem and small value results in under-learning problem [18]. *Xinhua and Ming* [19] hybridized Particle Swarm Optimization (PSO) algorithm and LSSVM, and proposed a PSO-LSSVM model to predict the deformation on surrounding rocks of underground caverns. Zhu [20] used PSO-LSSVM to measure key variables on-line in fermentation process. The comparative studies show that Grey-Wolf Optimization (GWO) algorithm has overall best performance in terms of search efficiency and convergence speed for finding a global optimum solution as compared to PSO, ABC, FFA, CS, BA, FPA, GSA, DE, EP and ES [21,22]. Hence, in this work, GWO is used to get optimum parameters of proposed LSSVM prediction model. It is notable to mention “No Free Lunch” (NFL) theorem here, which proves that an optimization algorithm works well in some specific optimization problems, but same algorithm is not successful in other set of optimization problems [23]. In our case, GWO provides best results that fulfill our requirements.

Many studies proposed different machine-learning-based prediction models for MPC to predict future outputs. A generalized predictive control (GPC) scheme is proposed to control concentration of bacteria by using a linearized PSO-LSSVM model [24]. However, linear models show limited control performance because industrial fermentation processes generally exhibit complex and severe non-linear behavior. Furthermore, if a non-linear model is used, the optimization problem becomes a non-convex problem, which is solved by using Non-linear Programming (NP) method. Conventionally, NP is involved in computationally expensive step of determining the hessian matrix and its inverse [25]. In addition, these solutions are highly dependent on the selection of initial point value and can easily fall in local optimal region (solution) [26]. The biggest challenge in designing an NMPC is to find an algorithm that minimizes a cost function in real time. The cost function is usually non-convex, high-dimensional with complex and non-linear constraints [27].

This work employs a novel derivative-free approach to solve non-linear and non-convex rolling optimization problem in NMPC to control product concentration of l-Lysine fermentation process. To the best of our knowledge, GWO has not been applied to solve rolling optimization problem in NMPC for control problems in fermentation process. GWO has fast convergence speed, involves lesser operators in computations and requires a few adjustable parameters [28]. These properties make GWO an ideal candidate to solve a non-linear, non-convex optimization problem of NMPC in real time. Furthermore, the performance of NMPC is highly dependent on the accuracy of the prediction model. Thus, constructing an accurate prediction model is a crucial step. The proposed non-linear GWO-LSSVM prediction model is employed in NMPC for prediction of future output values. In addition, GWO is imposed to solve rolling optimization problem in NMPC as Chen [29] designed a NMPC based on PSO to control greenhouse temperature. In this way, a non-linear MPC is designed which easily incorporates a non-linear prediction model and solves a non-convex optimization problem in real time. The final results of GWO-LSSVM prediction model and non-linear GWO-NMPC control algorithm are compared with PSO-LSSVM and PSO-NMPC, respectively. The results show that the predicted values by GWO-LSSVM are very close to actual values as compared to PSO-LSSVM, and product concentration follows an optimal trajectory by employing real-time GWO-NMPC control strategy. Moreover, the results of GWO-NMPC surpass PSO-NMPC in terms of error tracking and adaptability.

The rest of the paper is structured as: Section 2 consists of materials and methods, which explains MPC basics, LSSVM model, GWO algorithm, proposed GWO-LSSVM prediction model, GWO-NMPC algorithm and experimental setup. Section 3 includes results and discussion. The paper is concluded in Section 4.

## 2. Material and Methods

### 2.1. Model Predictive Control (MPC)

**Figure 1:**
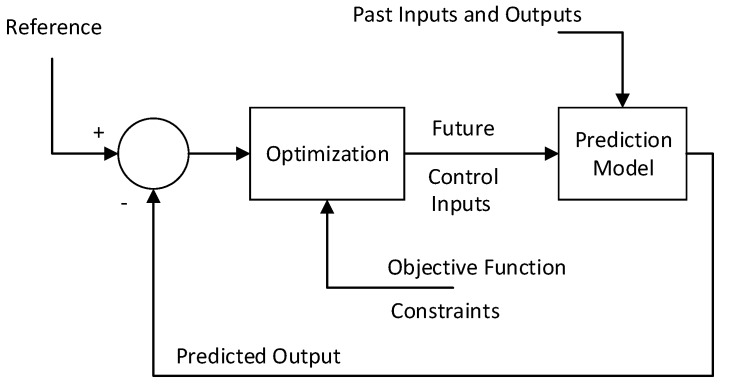
The Basic Structure of MPC.

Predictive control does not correspond to a particular control methodology but more precisely an abundant variety of control schemes, which exploits process model to obtain future control inputs that will force the system response to follow a desired response [30]. To accomplish the above mentioned objective, it minimizes a user defined objective function to obtain optimal future control inputs over a predefined prediction horizon $\left(N_{pred}\right)$ and control horizon $\left(N_{con}\right)$. Several MPC algorithms are different from each other because of the prediction models used to simulate the actual process and objective function that is used to solve the optimization problem. The basic structure of MPC is shown in Figure 1. The future control inputs are calculated by minimizing the error between the predicted output and a reference by solving an optimization problem. The general expression for an objective expression is as follows:

(1) $$\begin{array}{c} \begin{array}{c} \underset{\Delta u}{J(N_{pred},N_{con},u)}=\sum_{j=1}^{N_{pred}}Q_{out}\left(j\right)(y_{pred}\left(j+t|t\right)-y_{ref}{\left(j+t\right)}^{2}\\ +\sum_{j=1}^{N_{con}}R_{in}\left(j\right)({\Delta u(j+t-1))}^{2} \end{array} \end{array}$$

subjected to constraints:

(2) $$\begin{array}{c} \begin{array}{c} u_{min}\leq u\leq u_{max}\\ \Delta u_{min}\leq \Delta u\leq \Delta u_{max}\\ y_{min}\leq y\leq y_{max} \end{array} \end{array}$$

where *u*, Δu, $y_{pred}$, $y_{ref}$, $R_{in}$, $Q_{out}$ represent input, input increment, predicted output, desired reference, input penalization factor, error coefficient, respectively, and $u_{min}$, $u_{max}$, $\Delta u_{min}$, $\Delta u_{max}$, $y_{min}$, $y_{max}$ denote lower and upper bounds on control input, control increment, and control output, respectively. The steps included in MPC are:

1. Calculate output at the current time and calculate future outputs up to the prediction horizon $N_{pred}$.
2. Construct an objective function using predicted and reference values over a prediction and control horizon.
3. Minimize objective function to calculate optimal values of future inputs $U^{op}=u_{0}^{op},u_{1}^{op},\cdots ,u_{N_{con-1}}^{op}$.
4. Apply the first predicted input $u_{0}^{op}$ and discard all other future input values. Repeat the whole process at next sampling time t+1.

### 2.2. Least-Square Support Vector Machine (LSSVM)

To solve the computational complexity problem of SVM [31], Suykens proposed LSSVM [32]. In LSSVM, an equality constraint is introduced instead of inequality in SVM and a complex QP optimization problem is converted into the equations of linear system. In this way, the model decomposition and prediction problems can be solved efficiently. The basic principle is as follows:

Given *l* sample points for training, $\left\{\left(x_{i},y_{i}\right)|i=1,\cdots ,l\right\},x_{i}\in R^{n}$ is an input vector and $y_{i}\in R$ represents corresponding outputs. The approximation function in LSSVM is defined as:

(3) $$y\left(x_{i}\right)=\omega^{T}\varphi \left(x_{i}\right)+b$$

The optimization problem for regression is as follows:

(4) $$\begin{array}{c} \begin{array}{cc} min\;\;\underset{\omega ,\xi ,b}{J(\omega ,\xi )} & =\frac{1}{2}\omega^{T}\omega +\frac{g}{2}\sum_{i=1}^{l}\xi^{2}\\ s.t.\;\;\;\;\;\;y_{i}\left(x_{i}\right) & =\omega^{T}\varphi \left(x_{i}\right)+b+\xi ,\;i=1,\cdots ,l \end{array} \end{array}$$

where ω is a weight vector, $g\in R^{+}$ is penalty parameter, $\xi_{i}$ is an error variable, *b* is the deviation and φ(·) is mapping to a high-dimensional space. Lagrange method is used to optimize the above problems:

(5) $$\begin{array}{c} \begin{array}{cc} Ł(\omega ,\xi ,b,\alpha ) & =\frac{1}{2}\omega^{T}\omega +\frac{g}{2}\sum_{i=1}^{l}\xi^{2}-\sum_{i=1}^{l}\alpha_{i}\left(\omega^{T}\varphi \left(x_{i}\right)+b+\xi_{i}-y_{i}\right) \end{array} \end{array}$$

where $\alpha_{i}$ is a Lagrange multiplier. According to Karush–Kuhn–Tucker (KKT) conditions, the transformation to the linear equation is as follows [17]:

(6) $$\begin{array}{c} \begin{array}{c} \left(\begin{array}{cc} 0 & 1_{l}^{T}\\ 1_{l} & K+g^{-1}I_{l} \end{array}\right)\left(\begin{array}{c} b\\ \alpha \end{array}\right)=\left(\begin{array}{c} 0\\ y \end{array}\right) \end{array} \end{array}$$

where $y={\left[y_{1},y_{2},\cdots ,y_{l}\right]}^{T}$, $1_{l}={\left[1,\cdots ,1\right]}^{T}$, $I_{l}$ is $l_{t}h$ ordered unit matrix, $\alpha ={\left[\alpha_{1},\cdots ,\alpha_{l}\right]}^{T}$ and K is the kernel function matrix that satisfy Mercer’s conditions:

(7) $$\begin{array}{c} \begin{array}{c} K=\varphi {\left(x_{i}\right)}^{T}\varphi \left(x_{j}\right),\;\;\left(i,j\right)=1,\cdots ,l \end{array} \end{array}$$

In this paper, radial basis function is chosen as the kernel function because of its excellent performance and generalization ability, which is given as follows [33]:

(8) $$\begin{array}{c} \begin{array}{c} K=K\left(x,x_{i}\right)=\exp^{\frac{-|x-x_{i}{|}^{2}}{2\sigma^{2}}} \end{array} \end{array}$$

here σ is the kernel function width. Finally, the function of LSSVM is estimated as:

(9) $$\begin{array}{c} \begin{array}{c} y\left(x\right)=\sum_{i=1}^{l}\alpha_{i}K\left(x,x_{i}\right)+b \end{array} \end{array}$$

The prediction accuracy and generalization ability of LSSVM regression model strongly depends upon the penalty parameter ‘*g*’ and kernel width ‘σ’. So, these two parameters need to be optimized.

### 2.3. Grey-Wolf Optimization (GWO)

Mirajlili et al. proposed the GWO algorithm which imitates the social behavior of a grey wolf pack [22]. The grey wolves are divided into four categories namely *alpha, beta, delta* and *omega*. *Alpha* represents highest category and consists of leaders of the whole pack. *Alpha* wolves are responsible for making daily life decisions like hunting a prey, moving forward or stopping, sleep time and place. *Beta* group facilitates the *alpha* group in formulating these strategies and implementing commands on other lower categories. The third *delta* class is dedicated for fulfillment of above commands and controls *omega*. The lowest rank *omega* mainly obeys all instructions by superior departments. The hunting plan comprises of three steps: identifying and chasing the prey; encircling and harassing prey until it stops resilience; attacking on prey.

During the optimization process, it is assumed that the size of the grey wolf population is *n*, in an unknown *d*-dimensional search space. The position of grey wolves is denoted by $X_{wi}=\left[x_{i}^{1},x_{i}^{2},\cdots ,x_{i}^{d}\right]$. *Alpha* is considered to be the best fittest solution and its position is denoted by $X_{\alpha }$. Then, *beta* and *delta* are ranked as second, third best solutions and their locations are represented by $X_{\beta }$ and $X_{\delta }$, respectively. The remaining solutions represent *omega* class of pack.

The encircling strategy of the hunting process is mathematically modeled by the following equations:

(10) $$\vec{D}=\left|\vec{C}.{\vec{X}}_{p}\left(t\right)-{\vec{X}}_{w}\left(t\right)\right|$$

(11) $${\vec{X}}_{w}\left(t+1\right)={\vec{X}}_{p}\left(t\right)-\vec{A}.\vec{D}$$

where *t* indicates the current iteration, $\vec{A}$, $\vec{C}$ are coefficient vectors, ${\vec{X}}_{p}$ and ${\vec{X}}_{w}$ denote the position of the prey and wolf, respectively. $\vec{A}$ and $\vec{C}$ vectors are calculated as follows:

(12) $$\vec{A}=2\vec{a}.{\vec{r}}_{1}-\vec{a}$$

(13) $$\vec{C}=2.{\vec{r}}_{2}$$

where with an increase in number of iterations, $\vec{a}$ decreases linearly from 2 to 0. ${\vec{r}}_{1}$ and ${\vec{r}}_{2}$ are random numbers in range [0,1]. The hunting process is performed under the guidance of *alpha*. *Beta* and *delta* might also facilitate *alpha* in trapping a prey. So top three departments (*alpha, beta, delta*) have the best information (best solution) about the prey. These best solutions found so far are saved iteratively and other search agents (*omega*) are forced to follow and update positions according to these best positions. The mathematical equations that encapsulate all the above scenario are as follows:

(14) $${\vec{D}}_{\alpha }=\left|{\vec{C}}_{1}.{\vec{X}}_{\alpha }-\vec{X}\right|$$

(15) $${\vec{D}}_{\beta }=\left|{\vec{C}}_{2}.{\vec{X}}_{\beta }-\vec{X}\right|$$

(16) $${\vec{D}}_{\delta }=\left|{\vec{C}}_{3}.{\vec{X}}_{\delta }-\vec{X}\right|$$

(17) $${\vec{X}}_{1}={\vec{X}}_{\alpha }-{\vec{A}}_{1}.{\vec{D}}_{\alpha }$$

(18) $${\vec{X}}_{2}={\vec{X}}_{\beta }-{\vec{A}}_{2}.{\vec{D}}_{\beta }$$

(19) $${\vec{X}}_{3}={\vec{X}}_{\delta }-{\vec{A}}_{3}.{\vec{D}}_{\delta }$$

(20) $$\vec{X}\left(t+1\right)=\frac{{\vec{X}}_{1}+{\vec{X}}_{2}+{\vec{X}}_{3}}{3}$$

### 2.4. GWO-LSSVM Prediction Model

**Figure 2:**
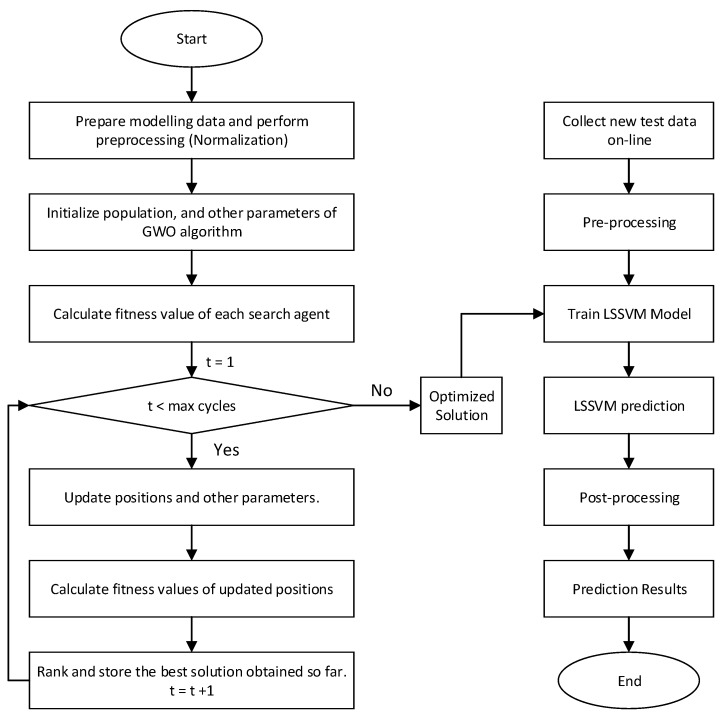
GWO-LSSVM prediction model.

The parameters ‘*g*’ and ‘σ’ of the LSSVM model play a critical role in the prediction accuracy. A very large value of penalty parameter ‘*g*’ would lead to remarkably high accuracy on training data but less accuracy on test data, while low value of ‘*g*’ makes the model less functional which results in poor performance [18]. In addition, an excessively large value of kernel factor ‘σ’ inflicts overfitting problem and small value results in under-learning problem. The kernel width ‘σ’ defines the effect of a single training example on other examples. Hence, there is a need to choose the values of LSSVM model parameters ‘*g*’ and ‘σ’ carefully. Researchers have used different optimization algorithms to select the optimum values of critical parameters of regression models, such as the PSO algorithm [24]. In this study, an efficient metaheuristic GWO algorithm is proposed to find best suitable parameters of LSSVM prediction model as shown in Figure 2. The steps of GWO-LSSVM are as follows:

Step 1: Prepare train, test, cross-validation data and perform pre-processing (normalization). Define number of search agents, maximum iterations, dimension of parameters to be optimized, lower and upper bounds.
Step 2: Randomly initialize *alpha*, *beta*, *delta* and *omega* positions, and $\vec{a}$, $\vec{A}$, and $\vec{C}$. Train LSSVM model on training data using these positions as ‘*g*’ and ‘σ’ value.
Step 3: Calculate fitness value of each search agent position. The fitness value corresponds to prediction accuracy of trained model on cross-validation data, which is calculated using user defined fitness function. In this study, *RMSE* is used as a fitness function given in Equation (22).
Step 4: Update the positions using Equations (14)–(20) and $\vec{A}$, $\vec{C}$ and $\vec{a}$ using Equations (12) and (13).
Step 5: Calculate again the fitness value of all updated positions.
Step 6: Rank and store the best solution obtained so far using fitness value. Repeat from step (4) to step (6) until maximum cycles are reached.
Step 7: Train again LSSVM model with best solution obtained from above steps and check the prediction accuracy on new test data to verify again model functionality.

### 2.5. GWO-NMPC Control Algorithm

The proposed GWO-LSSVM prediction model is employed in NMPC for prediction of future output values. Furthermore, the optimization problem in NMPC is solved by using GWO algorithm. Hence, a GWO-based non-linear MPC control strategy is used to achieve the objective of this study. GWO can incorporate the constraints on input, input increment and output value. The fitness function in GWO is replaced by the defined objective function. In this case, the position of the pack in GWO denotes the future control increments (penalty factor and kernel width in case of LSSVM optimization). GWO algorithm optimizes the objective function and finds optimum values of control increment Δu. The steps of GWO-NMPC are as follows:

Step 1: Control input variables, output variable and reference trajectory are defined.
Step 2: The constraints on inputs, input increments and outputs are defined.
Step 3: The control objective is accomplished by using an objective function as in Equation (1).
Step 4: In objective function, the predicted output ‘$y_{pred}$’ is estimated by using proposed GWO-LSSVM model.
Step 5: For each sampling interval, GWO optimizes the objective function and calculates the optimum values of control input increment Δu.
Step 6: The future control inputs are calculated by using following equation:
  (21) u(t+1)=u(t)+Δu(t+1)
  where *t*, *u*, Δu represent current sampling time, control input and control increment, respectively.
Step 7: Finally, calculated input is applied to the process and output feedback strategy is employed.

**Figure 3:**
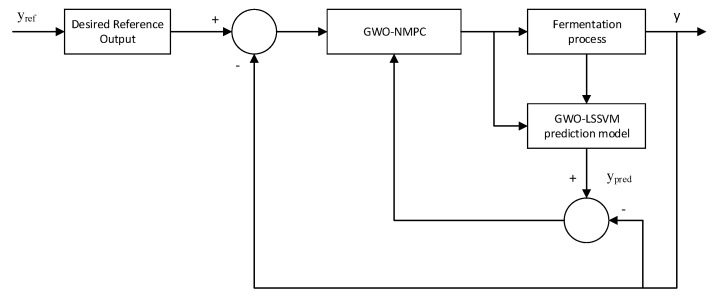
GWO-LSSVM-NMPC to control l-Lysine product concentration.

The final GWO-based non-linear MPC using a GWO-LSSVM prediction model control scheme for controlling the product concentration in l-Lysine fermentation process is shown in Figure 3. The prediction error is corrected on-line using output feedback.

### 2.6. Experimental Setup

The experiment of l-Lysine fed-batch fermentation was carried out at the control system platform of Jiangsu University. The RT-100L-Y fermenter model was used to perform this experiment. To make the experiment close to the actual production process, the experimental process was designed as follows:

1. In a 30 L mechanical stirring fermenter, fed-batch fermentation was conducted. The environmental parameters and physical parameters in the fermentation process were collected in real time by a digital measurement and control system composed of ARM development platform, and transmitted to the industrial control computer in the control room via a serial communication line. The time period for every batch was 72-h and the sampling time period was 15 min. The auxiliary inputs (such as temperature *T*, pH, agitation speed rate $u_{1}$, dissolved oxygen $D_{o}$, air flow rate $u_{2}$ and acceleration rate of ammonia flow $u_{3}$) were collected in real time. The key variable product concentration ‘P’ was sampled after every 2-h and tested in laboratory off-line. After this, the key biochemical variable was transformed from 2-h sampled data to 15 min sampled data (consistent with the number of auxiliary inputs data) in MATLAB using the “spline” interpolation function *interp1* (https://www.mathworks.com/help/matlab/ref/interp1.html). P was determined by the modified ninhydrin colorimetric method, i.e., 2 ml of the supernatant and 4 ml of the ninhydrin reagent were mixed and heated in boiling water for 20 min. The absorbance at 475 mm was measured by a spectrophotometer after cooling and obtained by checking the standard l-Lysine curve. These inputs represent the inputs ‘*x*’ in Equations (3)–(9). In addition, the product concentration ‘P’ represents the output ‘*y*’ in Equations (3)–(9). A non-linear mapping function is estimated using LSSVM between these inputs and output.
2. Ten batches were used for testing the modeling competence of the GWO-LSSVM method. The initial conditions between batches were set differently and the feeding strategy was also changed to enhance the differences between batches. The pressure of the fermentation tank was set to 0 ∽ 0.25 MPa, the temperature of fermentation was adjusted to 0 ∽ 50 °C ± 0.5 °C and the dissolved oxygen electrode was calibrated for the reference reading when the stirring motor was rotating at 400 rpm.

### 2.7. Performance Evaluation Metrics

To evaluate the accuracy of prediction model, statistical measures such as *Root mean square error (RMSE)*, *Mean absolute error (MAE)* and *Mean absolute percentage error (MAPE)* are used.

(22) $$RMSE\left(V_{actual},V_{pred}\right)=\sqrt{\frac{1}{T}\sum_{i=1}^{T}{\left(V_{actual}-V_{pred}\right)}^{2}}$$

(23) $$MAE\left(V_{actual},V_{pred}\right)=\frac{1}{T}\sum_{i=1}^{T}\left|V_{actual}-V_{pred}\right|$$

(24) $$MAPE\left(V_{actual},V_{pred}\right)=\frac{1}{T}\frac{\sum_{i=1}^{T}\left|V_{actual}-V_{pred}\right|}{V_{actual}}$$

where $V_{pred}$, $V_{actual}$ and *T* represent predicted, actual and total number of output values, respectively.

## 3. Results and Discussion

At first, the data are normalized in the range [−1 1]. Six batches are selected randomly to train the GWO-LSSVM model. Further two batches are selected for cross-validation step (off-line training and correction of model). After off-line validation of prediction model, the model is tested using optimized values of LSSVM parameters on two new batches of data to estimate the product concentration on-line. To find the optimum values of parameters of LSSVM, the parameters of GWO are adjusted as search agents $N_{max}=30$, maximum iteration $I_{ter}=100$, dimension dim=2, lower bound lb=[1,0.001] and upper bound ub=[10,000,0.1].

### 3.1. GWO-LSSVM Results Analysis

LSSVM parameters are optimized using GWO optimization. The wolf’s position in 2-dimensional search space (2D = number of LSSVM parameters to be optimized) represents LSSVM parameter values. The best position of alpha wolf denotes optimal parameters of LSSVM. *RMSE* is used as an objective function given in Equation (22). It defines the fitness of the solution (best position). The parameter values that minimize this objective function are selected as optimal solution which represent best position of *alpha* wolf. After selecting the optimal parameters obtained through GWO, LSSVM prediction model is established using these optimal parameters (GWO-LSSVM).

**Figure 4:**
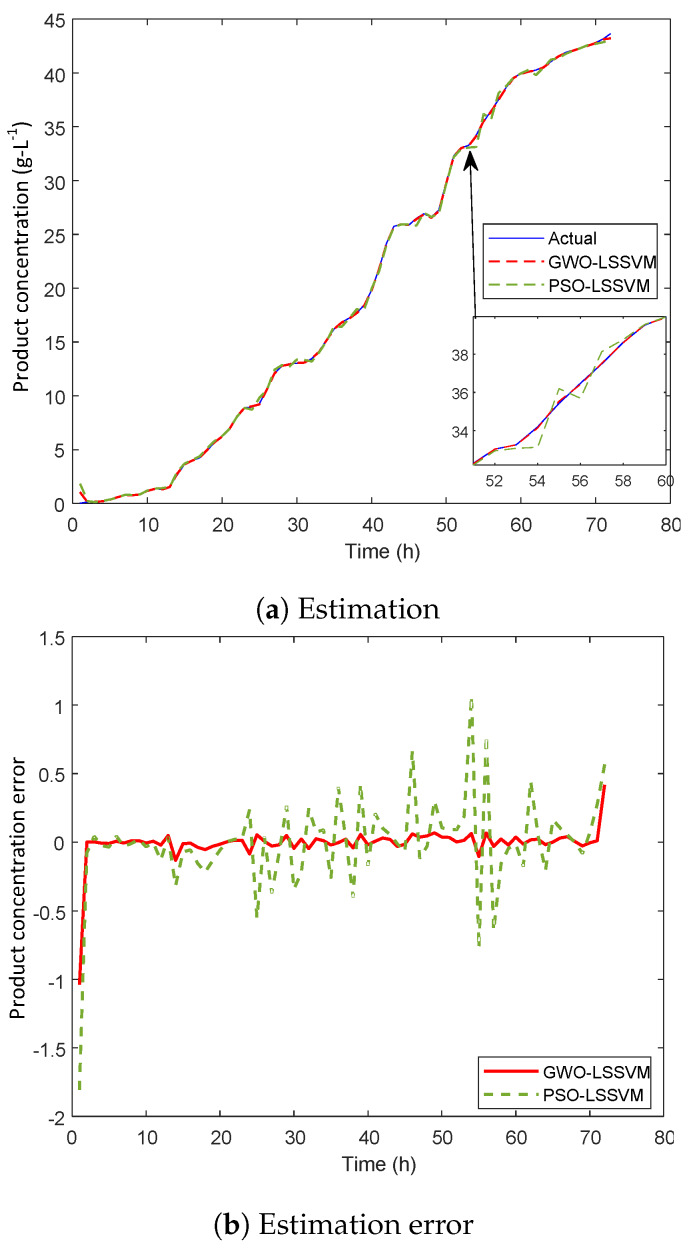
Product concentration prediction and error curve.

The proposed GWO-LSSVM is exploited as a prediction model and results are compared with PSO-LSSVM to show the effectiveness of GWO-LSSVM prediction model. PSO-LSSVM is the widely used prediction method in biological fermentation processes. For example, PSO-LSSVM is used to predict inulinase concentration in Pichia pastoris fermentation process [34]. Zhu [20] used PSO-LSSVM to measure key variables in Penicillin fermentation process. The actual and predicted curves by proposed GWO-LSSVM and PSO-LSSVM are shown in Figure 4a. The results clearly show that the GWO-LSSVM prediction model is capturing the future variation trends accurately. Furthermore, the difference between actual and predicted value is plotted in Figure 4b to visualize error more clearly. We can see that the amplitude of error spikes for GWO-LSSVM is much lower than that of PSO-LSSVM. In addition, three statistical performance evaluation metrics are presented in Table 1. The *RMSE* value of GWO-LSSVM is approximately 61 % less than PSO-LSSVM. Similarly, the difference in the values of *MAE* and *MAPE* is approximately 77 % and 43 %, respectively. It is evident from the results of these statistical measures that GWO-LSSVM perform better than PSO-LSSVM. Although PSO has been very successful in many applications, it is more vulnerable to getting stuck at local minimum. According to NFL theorem, there is no single existing optimization algorithm that would be applicable in all kind of optimization problems. In each optimization problem, the optimization algorithm encounters a different unknown search space. The success of optimization algorithms depends upon the basic strategy of mathematical models that tries to avoid the local optimal solutions. In our case, the GWO-based LSSVM prediction model is more competent as compared to PSO-LSSVM. The prediction error is negligible and almost all error values lie in range [−0.1 0.1], which is satisfying the controller requirement employed to control the product concentration. Thus, the proposed GWO-LSSVM prediction model is the best choice to be used as a process model in current non-linear MPC.

### 3.2. GWO-NMPC Results Analysis

The proposed non-linear MPC uses the search efficiency, local and global search ability of GWO for manipulating the input variables to optimize the future behavior of product concentration in l-Lysine’s fermentation process. Dissolved Oxygen ‘$D_{o}$’ and ‘pH’ have a strong influence on product concentration. Thus, by monitoring the product concentration and according to its required optimum growth, $D_{o}$ and pH are manipulated using agitation rate ‘$u_{1}$’, airflow rate ‘$u_{2}$’ and ammonia flow rate ‘$u_{3}$’. Hence, three manipulated input variables are agitation rate ‘$u_{1}$’, airflow rate ‘$u_{2}$’, ammonia flow rate ‘$u_{3}$’. The controlled output ‘*y*’ is product concentration. The initial substrate concentration and temperature values are 150 g L${}^{-1}$ and 32 °C, respectively. The objective function in Equation (1) is used as a fitness function of GWO, such as *RMSE* is used in the optimization problem of the LSSVM parameters. The error coefficient and input penalization coefficient are usually selected as a constant value. In this work, the value of error coefficient is selected as $Q_{out}=1$ and input penalization coefficient is $R_{in}=0.001$. The constraints on inputs, input increments and output are defined as:

(25) $$\left\{\begin{array}{c} 316\leq u_{1}\leq 345\\ 0.1\leq u_{2}\leq 1.6\\ 51\leq u_{3}\leq 110 \end{array}\right.$$

(26) $$\left\{\begin{array}{c} -15\leq \Delta u_{1}\leq +15\\ -1\leq \Delta u_{2}\leq +1\\ -30\leq \Delta u_{3}\leq +30 \end{array}\right.$$

(27) 0≤y≤45

The initial inputs are selected as $U_{10}=326$, $U_{20}=0.1$ and $U_{30}=88$. These initial input values are used to calculate the first future optimal input values according to Equation (21). The prediction and control horizon are defined as $N_{pred}=N_{con}=1$. These parameter values are selected after extensive simulations. The increase in prediction and control horizon values have no significant difference on performance in our problem. Furthermore, our requirement is satisfied with these minimum horizon values. The computational cost increases with the increase in value of these horizons. However, these values can be increased that depends on the objective of controlling process to acquire desired response. GWO-LSSVM prediction model predicts the future output value ‘$y_{pred}$’ in defined objective function as in Equation (1). As GWO-LSSVM prediction model is non-linear, and objective function consists of non-linear constraints on manipulated inputs (agitation speed rate $u_{1}$, air flow rate $u_{2}$ and acceleration rate of ammonia flow $u_{3}$), so the optimization problem to solve this objective function for optimal future inputs ($u_{1}$, $u_{2}$ and $u_{3}$) becomes a non-convex and non-linear optimization problem. Now, GWO solves this optimization problem to find the future optimal values of these manipulated inputs at each sampling time. Here, a 3-dimensional search space is defined (3D = number of optimal future inputs to be determined). The wolves position in 3 *dim* space represents future optimal increment in input values ($\Delta u_{1}$, $\Delta u_{2}$ and $\Delta u_{3}$). The optimal future input increments that minimize the defined objective function (best *alpha* wolf position) are calculated in advance at each sampling instant for next sampling time. These optimal increments in inputs are used to determine the optimum values of future input rates using Equation (21). Then, these optimized input rates are applied to acquire the desired response. At next sampling point, the whole process is repeated for upcoming sampling instant to achieve the required objective.

#### 3.2.1. Hypothetical Case Study

The proposed GWO-based predictive control scheme is compared with the PSO-based predictive control method. GWO-LSSVM is used as a prediction model, in proposed NMPC, to predict product concentration such as PSO-LSSVM is exploited as a prediction model in a predictive control method to predict and control bacteria concentration [24]. Similarly, PSO is used in a study to solve the rolling optimization problem in a predictive control scheme to control substrate concentration and LSSVM is used as a prediction model [35].

**Figure 5:**
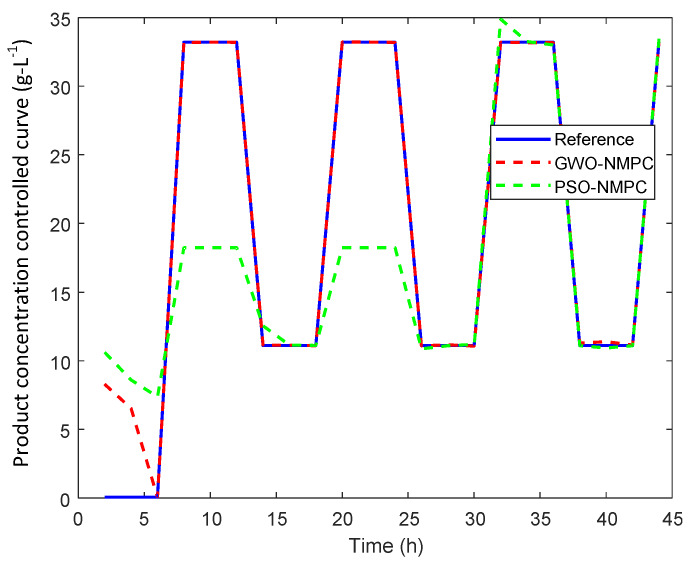
GWO-NMPC controlled product concentration output with hypothetical reference.

**Figure 6:**
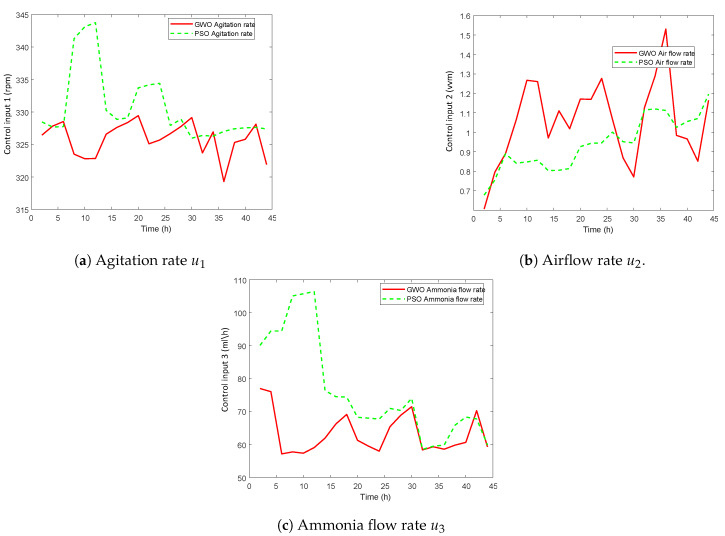
GWO-NMPC controlled inputs with hypothetical reference

Initially, a hypothetical reference signal with sharp periodic step changes is applied to validate the robustness and adaptability of GWO-NMPC for the first 44 hours, as shown in Figure 5. The simulation results are compared with PSO-NMPC. Although this kind of phenomenon is not realistic in fermentation process because fermentation is a slow time varying process, yet it proves the robustness and adaptability of the proposed control scheme. The corresponding manipulated input variables are shown in Figure 6a–c.

#### 3.2.2. Real Case Study

**Figure 7:**
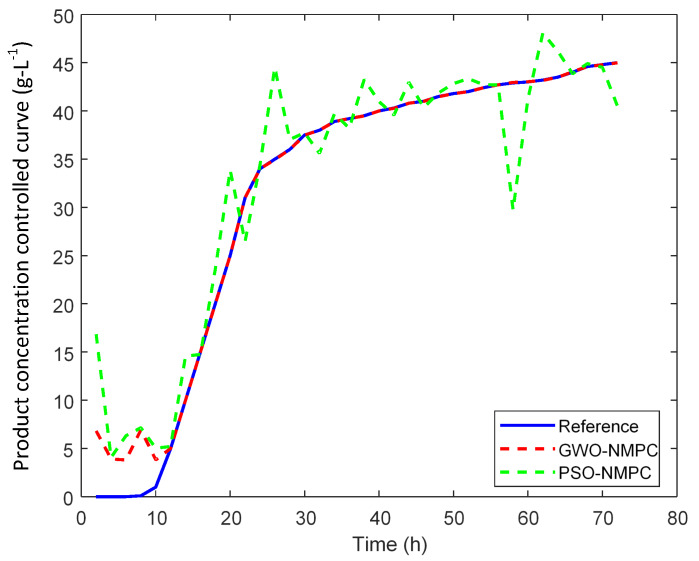
GWO-NMPC controlled product concentration output with optimal reference.

**Figure 8:**
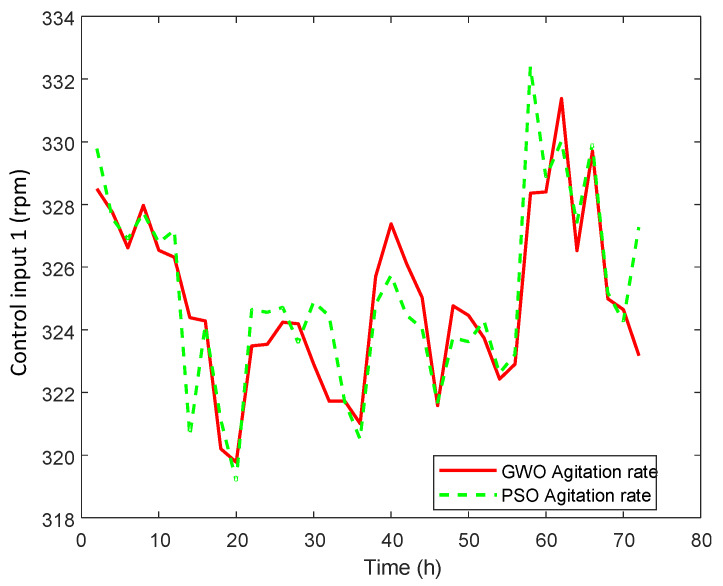
GWO-NMPC controlled agitation rate $u_{1}$ with optimal reference.

**Figure 9:**
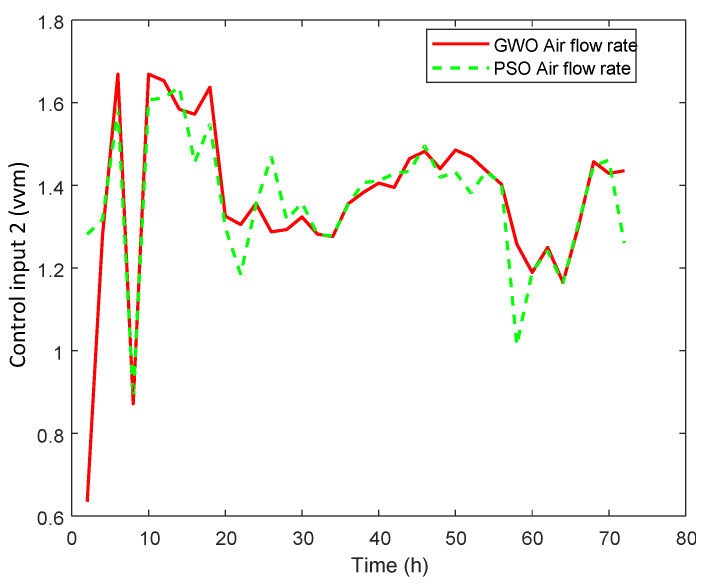
GWO-NMPC controlled airflow rate $u_{2}$ with optimal reference.

**Figure 10:**
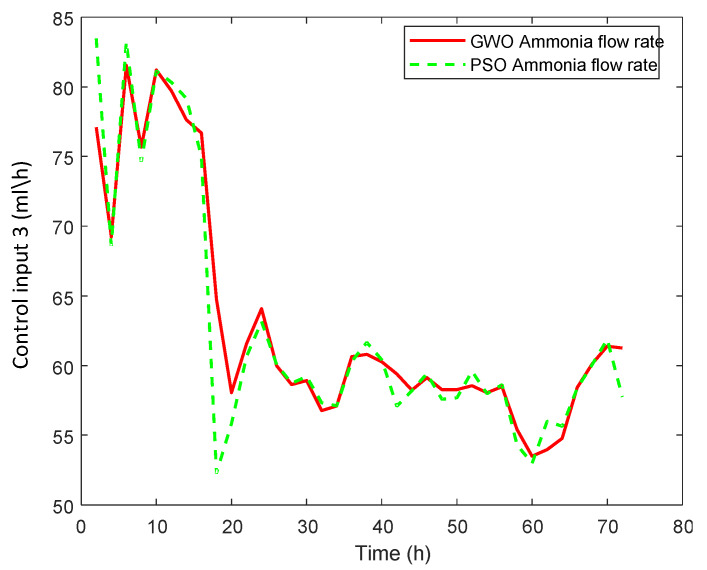
GWO-NMPC controlled ammonia flow rate $u_{3}$ with optimal reference.

An optimal selected trajectory as a reference is applied and the results are shown in Figure 7. The corresponding manipulated inputs are shown in Figure 8, Figure 9 and Figure 10. We can see that the proposed method enforced the desired response and process follows this optimal trajectory accurately. GWO possesses fast convergence speed, excellent local, and global search ability. GWO is successful in handling non-linear constraints to solve a non-convex optimization problem for optimal future input values in real time and provides best global solution as compared to PSO. In addition, GWO-NMPC outperforms PSO-NMPC in terms of prediction accuracy, control precision and near-to-accurate tracking ability. The overall error in GWO-NMPC is negligible as compared to PSO-NMPC. The success of NMPC highly depends upon accuracy of process model used for output prediction. GWO-LSSVM encompasses precise dynamics and non-linear behavior between inputs and output of the l-Lysine fermentation process. This results in efficient performance of the proposed GWO-NMPC. Due to such optimal and controlled conditions, the osmotic stress or catabolic repression of bacteria is avoided successfully, and the final yield is increased by 25 % approximately. Therefore, it shows that the machine-learning-based prediction models and predictive control schemes are effective for control and optimization of complex non-linear industrial processes.

## 4. Conclusions

A non-linear MPC by exploiting a machine learning-based prediction model is proposed to control product concentration in real time. LSSVM prediction model, which requires very few input and output samples for training, is deployed in NMPC. The traditional experience and trail-error-based method to select optimal value of LSSVM parameters is replaced by employing a novel metaheuristic GWO algorithm. Thus, real-time identification problem is solved by proposed GWO-LSSVM prediction model and it eliminates the need for an accurate kinetics mathematical model. Furthermore, to cope with the non-linear, non-convex and complex constraints-based optimization problem in NMPC, a gradient free approach is proposed. A novel GWO-based algorithm is established to compute optimum future input values by minimizing a cost function in real time. The proposed GWO-NMPC control scheme provides an efficient way to deal with complex, non-linear and dynamic systems. In future, we are interested to extend this work to control and optimize further key variables in fermentation process by designing multi-input and multi-output models.

## Figures and Tables

**Table 1:** RMSE, MAE and MAPE comparison.

Model	RMSE	MAE	MAPE
GWO-LSSVM	0.136918	0.047230	0.703616
PSO-LSSVM	0.355483	0.212182	1.244831

## Abbreviations

The following abbreviations are used in this manuscript:

NMPC	Non-linear Model Predictive Control
SVM	Support Vector Machine
LSSVM	Least-Square SVM
GWO	Grey-Wolf Optimization
PSO	Particle Swarm Optimization
ANN	Artificial Neural Network
QP	Quadratic Programming
ABC	Artificial Bee Colony
CS	Cuckoo Search
FFA	Firefly Algorithm
BA	Bat Algorithm
FPA	Flower Pollination Algorithm
GSA	Gravitational Search Algorithm
DE	Differential Evolution
EP	Evolutionary Programming
ES	Evolution strategy
NFL	No Free Lunch
GPC	Generalized Predictive Control
NP	Non-linear Programming
KKT	Karush–Kuhn–Tucker conditions
NP	Non-linear Programming
RMSE	Root Mean Square Error
MAE	Mean Absolute Error
MAPE	Mean Absolute Percentage Error
ml	Milliliter
mm	Millimeter
rpm	Revolutions per minute
MPa	Megapascal
vvm	Volume per Unit per Minute
